# Strategies for Growing Large-Scale Mycelium Structures

**DOI:** 10.3390/biomimetics7030129

**Published:** 2022-09-11

**Authors:** Jonathan Dessi-Olive

**Affiliations:** MycoMatters Laboratory, University of North Carolina at Charlotte (UNCC), Charlotte, NC 28223, USA; jdessiolive@uncc.edu; Tel.: +1-952-334-1775

**Keywords:** mycelium, myco-materials, myco-fabrication, sustainable buildings, sustainable structures, architectural design, structural design, material ethics

## Abstract

Fungi-based materials (myco-materials) have been celebrated and experimented with for their architectural and structural potential for over a decade. This paper describes research applied to assembly strategies for growing large building units and assembling them into efficiently formed wall prototypes. A major concern in the development of these two fabrication strategies is to design re-usable formwork systems. La Parete Fungina demonstrates two undulating wall units standing side-by-side, each composed of seventeen myco-welded slabs. L’Orso Fungino revisits the in situ monolithic fabric forming of units that are repeated, stacked, and post-tensioned. Although the design and research presented in this paper focuses on overcoming the challenges of growing large-scale building components, this work also touches on issues of accessibility and technology, economic and logistical systems needed for building-scale applications, and material ethics of energy and waste associated with emerging biomaterial production.

## 1. Introduction

Within Euro-centric traditions of architecture, the significance of a building is often tied to its permanence. The Pantheon in Rome, for example, is a nearly 2000-year-old cementitious dome structure, whose resilience to time elevates it to a monumental status. Notwithstanding the significance of cultural and economic factors associated with the need for permanent buildings and structures, must all buildings be assembled with the goal of being permanent? Globally, the lifespans of buildings are rapidly decreasing. The average lifespan of buildings in China was recently reported to be 34 years [1], and 25 years for residential buildings in Japan [2]. To great detriment, buildings are more than ever being demolished prematurely and yet, use materials that are manufactured with energy-intensive processes and are expensive or impractical to recycle. In the United States alone, the Environmental Protection Agency (EPA) reported there was 600 million tons of construction and demolition waste generated in 2018 [3]. Structural materials, including wood, and architectural metals, such as steel, copper, and brass, are valuable commodities that can be reused and recycled. However, in present-day architectural assemblies, these materials nearly ubiquitously inter-face with expanded foams, plastics, and resins, sometimes in irreversible composites. For example, wood is widely treated with synthetic resins and glues to increase its resistance to decay or structural performance.

Fossil-fuel-based materials are versatile and economical. They are used to create building products such as floor and wall finishes, furniture, conduits, structural reinforcements, insulation, and sealants, to name a few. From their manufacture to their end-of-life, synthetic materials require significant amounts of energy and produce emissions that are harmful to environmental and human health. Plastics, such as polyvinyl chloride (PVC), use a known carcinogenic monomer (vinyl chloride) in their production [4], and are often manufactured to be more ductile using phthalate plasticizers, a known class of toxins posing risks to the immune response, reproductive health, and embryonic development [5]. Particularly in Europe, sorting programs are improving, and assessments of recycling products, such as PVC from window frames [6], have demonstrated successful programs for those contexts. Still, only 3 percent of PVC is diverted from the waste stream in Europe [4]. Expanded polystyrene (EPS), commonly used as a packaging material, is fully recyclable, but due to its low density, the cost of transporting it to be recycled quickly outweighs the benefit if performed over long distances [7]. The EPA reports that only 0.6 percent of EPS waste produced in the United States is recovered [8]. While the championing of recycling has kindled examples of robust systems that produce high recycling rates in Germany and Singapore [9], the fate of most foams, plastics, and fossil-based composites is disposal in landfills, elimination through thermal incineration, or pyrolysis [10].

At a time when buildings can be expected to have short, non-permanent lifespans that commonly result in landfill disposal, new building materials are needed that can help challenge our traditional perceptions of significance and building permanence, rethink what materials we use to build, and gain awareness of where those materials go when we are finished with using them. Wood has recently been championed for its potential as a low-cost and affordable building material, but a labor shortage during the COVID-19 pandemic caused the cost of wood to increase by nearly four times [11], exposing the fragility of existing supply chains. In the face of material insecurity, there is a critical need to explore and test alternate low-energy and rapidly renewable building materials that contribute to circular material economies and lessen the impact of the architecture, engineering, and construction industries on climate change. Adopting new materials into the standards of contemporary and future construction is challenging, but necessary. Importantly, the way such new materials are used to design and build at the architectural scale cannot be assumed. Innovation is possible, and presenting physical demonstrations at the building scale is an important aspect of research needed to prove that an emerging material is viable for future building construction.

### 1.1. Mycelium Composite Materials

Fungi-based materials are among a class of biotechnologies showing promise in vastly offsetting the impact of the short lifespans of buildings in the modern era. In their most common form, lignocellulosic fibers sourced from agriculture or forestry material streams are bound together with an entangled web of *mycelia*, the root-like structures of fungi [12]. Commonly known as “myco-materials”, they are produced similarly to commercial mushroom farming, and can be composted at end-of-life. Myco-materials have become an international enterprise and are produced at an industrial scale. Companies such as Ecovative [13], Mycoworks [14], and Mogu [15] have explored their unique and variable properties to create products through different forms of production. Products finding commercial success include packaging materials [16,17,18], interior products such as lampshades and planters [19], and acoustical panels [15]. Mushroom leather products that serve as a sustainable alternative to animal leather are demonstrating increasing commercial success [14,20,21], and are created through the use of different solid- and liquid-state techniques [22].

Growing myco-materials involves propagating fungal hyphae (often from the phylum Basidiomycota) into a fibrous substrate for several days under correct environmental conditions until it forms a composite mass. Mycelium biomass is formally agnostic, having the capacity to be grown into nearly any shape by packing fibers inoculated with a living fungus into a formwork composed of a breathable non-cellulose-based material (usually plastic) to avoid the mycelium from permanently adhering to the mold. The limitations for growth are biological and environmental. Important precautions are proper sterility to avoid the contamination of unwanted organisms, access to food and nutrients, maximal darkness, and access to warm, humid air. Depending on the region, the fungal species being grown, and the scale of production, growth chambers may need to be actively controlled to maintain an optimal temperature and humidity, representing a likely demand for energy resources. A common issue myco-material growers face is the emergence of contaminants, sometimes dangerous molds, and other organisms that thrive in similar environmental conditions. Typically, the fibrous substrates into which mycelia are grown need to be steam-sterilized or pasteurized, which can also be prohibitively expensive due to the equipment and energy needed for such processing. Another important precaution that relates to design with myco-materials is that at certain thicknesses, mycelia do not grow sufficiently due to a lack of oxygen, presenting a chance for contamination.

Once fully grown, parts are typically actively dried to stop growth [23], resulting in a material that resembles expanded polyurethane or polystyrene foam with a flame spread resistance comparable to gypsum and low thermal conductivity. The numerous complexities associated with growing myco-materials make it difficult to control the associated material properties (whether mechanical, thermal, acoustical, or other) and are understood to be a reported average. Different combinations of mycelium strains and fibrous substrates yield varying properties of structural integrity, density, thermal conductivity, moisture resistance, and visual quality [24]. Studies have reported on mechanical qualities [25,26], the impact of moisture [27], acoustical properties based on mycelial growth [28], fire resistance [29], and their biodegradability [30], and their aesthetic capacities [31], among several others.

One of the most significant challenges of using mycelium in large-scale structural applications is that it is an inherently weak material (0.1–0.2 MPa of compressive stress on average without mechanical compaction) and assumed to work best in compression. Despite this limitation, myco-materials are also very lightweight, giving them advantageous strength-to-weight ratios compared to concrete. This suggests that through advantageous material placement large-scale and even long-span structures are possible. In the last decade, several large-scale pavilion structures have demonstrated the potential of myco-materials to be used for building structures. An important distinction must be determined between those which use mycelium in a load-bearing capacity, and those which use the material as a surface or cladding application. Pavilions such as “Shell Mycelium” in India [32], the “Living Pavilion” in the Netherlands [33], and the pavilion at the Rensselaer Polytechnique Institute, Troy, NY, USA [34], used mycelium cladding panels or units over wooden frame structures. Ecovative used mycelium panels as the insulation of a tiny house [35]. While these serve as examples of the building-scale use of myco-materials, they are definitively non-structural applications. Curiously, there has been little diversity in approaches to building with myco-materials, with fabrication techniques used to assemble myco-structures remaining canonically familiar to architecture and engineering. These include logical adaptations of assembly systems with bricks or blocks, monolithic castings, 3D printing-based, and hybrid techniques, which are described below.

### 1.2. Brick and Block Myco-Structures

The most common approach is based on the production of bricks or blocks grown in custom-made molds, actively dried in ovens, transported to the site and assembled, typically with the assistance of a temporary formwork and scaffolding structures. An early structural application of myco-materials was the “Myco-tectural Alpha” [36], a small catenary barrel vault built from bricks grown from reishi. The largest, and perhaps most widely publicized mycelium structure was the “Hi-Fi” [37], a 40-foot tower installation by David Benjamin and The Living in 2014, engineered by ARUP. The mycelium bricks sourced from Ecovative were stacked atop of a wood and steel supporting structure. The “MycoTree” exhibited at the 2017 Seoul Biennale [38] demonstrated how the structural capacity of mycelium can be exploited maximally by placing it in compression-only configurations. In each previous example, the structures were formed with the assumption that the material would only work in compression, with dome/vault, tower, and column structural forms dominating the literature. The masonry units themselves were grown in plastic formworks. Three-dimensional printing techniques for myco-materials have also been explored, with much attention being paid to the formulae of viscous living pastes to be extrude with techniques adopted from digital ceramics [39]. Unit-based column structures have been demonstrated by teams in Europe at Lund University, Lund, Sweden [40], and by Blast Studio, London, the UK [41]. Among the numerous exciting prospects of 3D printing myco-materials, a significant benefit is that custom-designed building units can be produced without needing a plastic formwork.

### 1.3. Monolithic and Bio-Welded Myco-Structures

Though much weaker and lighter than concrete, grow-in-place monolithic mycelium techniques can inherit many of the advantages (and challenges) of cast-in-place concrete techniques, including the use of traditional board, plank, sheeting, and flexible fabric formwork techniques. Without some means of aeration, beyond a certain thickness (150 mm or so), there is a risk that the fungi die prematurely from a lack of oxygen. Beyond assemblies of discrete element techniques, other research has focused on stereotomic approaches and monolithically growing large colonies of myco-materials in situ.

#### 1.3.1. Monolithic Myco-Structures

Monolithic mycelium requires the design and fabrication of complex formworks that permit the fungi to fully grow. Due to such challenges associated with the cultivation of large volumes of live myco-materials and the constructing of formworks to facilitate such growth, very little work on monolithic mycelium has been accomplished in the context of architecture and structural design. In 2016, a master’s of science thesis on civil engineering at Miami University, in Coral Gables, FL, USA [42] suggested analytical methods for mycelium-based monolithic domes, but did not validate them through physical means. At a small scale, Dutch artist Eric Klarenbeek demonstrated structural monolithic growth [43] in combination with 3D printing to create furniture. Ecovative experimented with monolithic mycelium and exhibited a chair in 2018 [44] that used a proprietary process that aerated the growing colonies of myco-materials, allowing them to be grown at greater thicknesses. A dissertation from the University of Newcastle in Newcastle upon Tyne, the UK, explored the potential of monolithic mycelium chair structures [45] grown in a conventional plastic formwork. Another interesting application of monolithic mycelium was a functional canoe [46] that was over 2 m long, grown by a student at Wayne State College in Wayne, NE, USA, in 2020.

**Figure 1:**
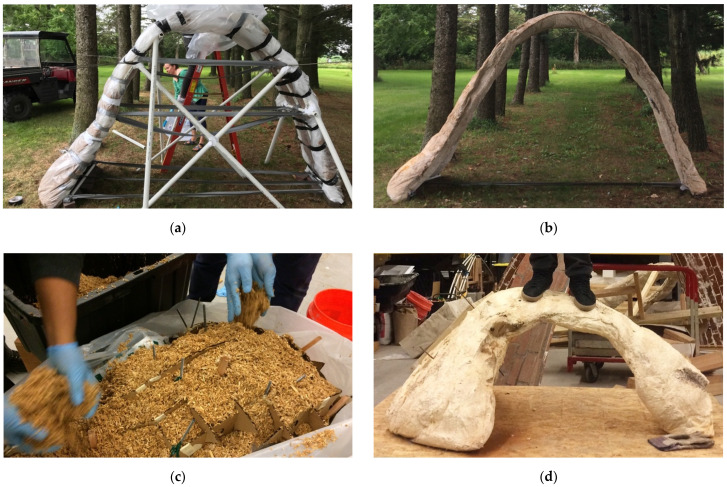
(**a**) Formwork for the “Mycoarch” composed of active bent PVC and plastic sheeting; (**b**) completed arch (late 2017, since renamed the “Diamond A Arch”), which collapsed due to inaccurate form and a myco-material matrix that had not sufficiently dried; (**c**) packing the internal reinforcing for the “Thick and Thin Arch” composed of recycled cardboard; (**d**) complete “Thick and Thin Arch” (early 2018) held seventy-five kilograms. Photos by the author.

Beyond these examples in product and furniture design, very few examples of architectural structures have been attempted. A series of three prototype structures was previously presented by the author of this paper [47], proving that grow-in-place monolithic mycelium structures were feasible through novel constructive approaches. Two arch structures (Figure 1) brought to light crucial considerations for successfully growing monolithic mycelium structures. First, the external formwork must be strong enough to support the weight of a wet substrate while maintaining its precise form, it must be composed of removable non-cellulose materials, and must be sufficiently porous to allow promoting the mycelia to breathe. Second, internal reinforcing strategies are advisable to handle eccentric loadings and formal accuracy, and must be composed of a cellulose-based material to permit the mycelia to bind and grow through the reinforcing structure.

**Figure 2:**
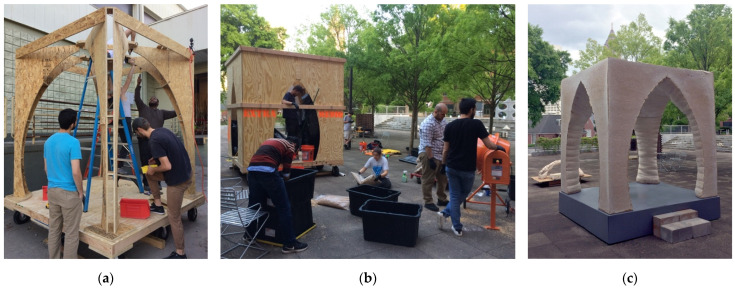
The Monolito Micelio, grown in early 2018 with students at Georgia Institute of Technology. (**a**) Construction of the wooden internal reinforcing; (**b**) in a manner resembling cast-inplace concrete, mycelilum composite materials were processed on-site with water and nutritional additives and immediately packed into the plywood and geo-textile formwork; (**c**) finished structure, used as a stage and pavilion for a choir performance and exhibited at the School of Architecture. Photos by the author.

A third prototype structure, called the Monolito Micelio (Figure 2), was an architectural-scale monolithic mycelium structure, grown in early 2018 from a one-ton colony of mycelium-stabilized hemp procured from Ecovative. The structure was designed and executed in the context of a graduate research seminar at the Georgia Tech School of Architecture. The vaulted pavilion was a critical response to the observed monotony of brick/block-based myco-fabrication methods and built upon the constructive principles of structures before it. The pavilion demonstrated that myco-materials could inherit fabrication logics from cast-in-place concrete techniques, including traditional board formwork and flexible fabric formwork techniques. Importantly, the structure showed that much more work was needed to uncover new and previously unimaginable construction logics that go beyond the architectural cannon of traditional materials.

**Figure 3:**
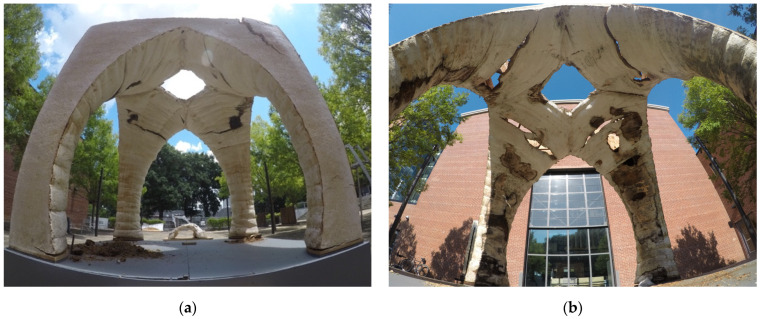
Decay of the Monolito Micelio. (**a**) Cracking and decay of the structure after three months caused by expansion and contraction of the material matrix against the internal reinforcing structure; (**b**) cracking, decay, and infestation of the structure after four months. Photos by the author.

The success of the project was also met with numerous failures, which provided the grounds for such a future inquiry. Notably, as part of a super-structure, myco-materials are highly susceptible to expansion and contraction in the face of external elements, making them unsuitable for external use, unless for temporary structures where the lifespan of the structure is understood to be short. Temperature swings and precipitation caused the material matrix of the Monolito Micelio to crack, decay, and become infested by other unfavorable organisms, including potentially dangerous mold (Figure 3). Furthermore, the materials used for the internal reinforcing system were much stronger and rigid than the myco-materials, which further exacerbated the cracking and decay of the structure.

**Figure 4:**
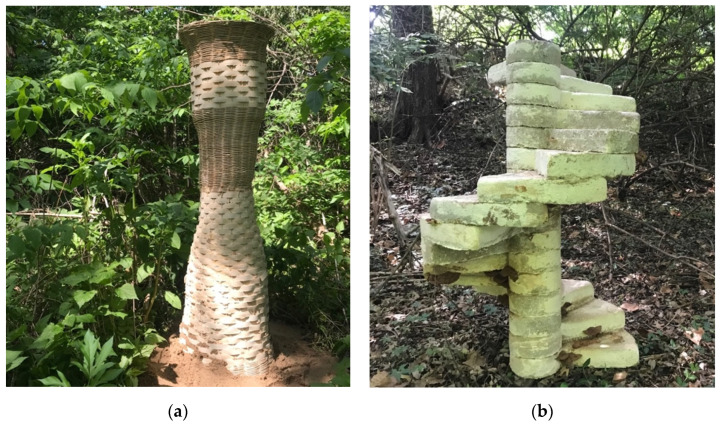
Monolithic and myco-welded structures grown by the author and students at Kansas State University in spring 2021, shown in their final installation sites. The structures were both larger than the available resources for actively drying the structures to stop growth, resulting in the emergence of fruiting bodies on the structures. (**a**) Two-meter-tall woven monolithic mycelium column; (**b**) half-scale myco-welded staircase with visible fruiting bodies that resulted from the two-stage growing process inherent to the myco-fabrication technique. Photos by the author.

While, in many regional contexts, there are minor active energy inputs needed to grow myco-materials, their reliance on plastics and molds that have limited reusability presents an ethical dilemma. For example, the plastic-lined plywood and woven nylon fabric formwork system used for the Monolito Micelio was a waste byproduct that resulted in land-fill disposal. The issue of formwork resulting in waste is an issue that has since been taken up by researchers interested in monolithic mycelium. A prototype structure by the multi-disciplinary collaboration in Europe called the FUNGAR project [48] provided early evidence that woven Kagome structures are an advantageous replacement for the polymeric in-situ formworks and molds typically needed to grow myco-materials. Such weaving crafts are globally ubiquitous, formally flexible, and often use natural lignocellulosic materials that are readily available. Such strong porous surfaces allow the fungi to breathe, provide a humid environment, and serve as a source of nutrition for the fungi. In contrast to plastic formworks, myco-weaves encourage mycelia to grow into the formwork and integrate into the biomass. More recently, the author of this paper grew a two-meter-tall monolithic mycelium column [49] along with students at Kansas State University that used basket weaving techniques. The woven formwork both participated in the visual expression of the column and potentially strengthened the assembly due to the deep bonds between the myco-materials and exoskeleton (Figure 4a).

#### 1.3.2. Bio-Welded Myco-Structures

An increasingly popular technique called “bio-welding”, or “myco-welding”, involves assembling structures with discreet living parts and growing them together into monolithic wholes. Myco-welding is challenging because it requires two stages of growth. First, individual units are grown from loose inoculated substrates in molds. Second, assemblies of living units are kept in an intended formal configuration for several days, while maintaining necessary sanitary and environmental conditions. Drying and stopping the growth of large assemblies is also a challenge inherent to myco-welding large assemblies. If not completed quickly enough, fruiting bodies often grow on the structure (Figure 4b), which, depending on the application or context, may or may not be desirable. The technique has been demonstrated for small arch structures [50], furniture [51], for making monolithic blocks for use with robotic-controlled abrasive wire cutting [52], and a load-bearing half-scale spiral staircase recently grown by the author and their students [49]. At the large scale, the technique was demonstrated in the form of a triumphal arch at a short-term art installation in Europe [53].

### 1.4. Aims and Scope of This Research

The applied research described in this paper seeks to expand upon fabrication techniques using myco-materials, with the primary motivation being the excessive waste produced by contemporary construction practices. Among the numerous challenges and limitations associated with the application of myco-materials in architecture, this work focuses on overcoming (1) the challenge of cultivating large colonies of living myco-materials into precise forms and (2) the need for intuitive and re-usable formwork systems that reduce waste byproducts from growing and fabrication processes. The myco-fabrication strategies presented here were developed through the production of prototype structures that demonstrate growing large blocks of myco-materials and assembling them into efficiently formed wall structures. The prototypes share an underlying serpentine geometry deployed into assemblies that are categorically hybrids between monolithic and brick/block-based. One wall prototype demonstrates units created from myco-welded slabs, while the other revisits the in situ monolithic fabric forming of units that are repeated, stacked, and post-tensioned. Both structures were produced in academic contexts in collaboration with students from the University of Virginia (UVA) and Kansas State University (K-State), under the direction of the author. The prototypes were exhibited publicly in early 2022 at the Biomaterial Building Exposition (BBE) [54].

## 2. Context, Design, and Methods

The BBE gathered five teams of architect scholars from across the United States to develop and exhibit novel approaches for architectural-scale biomaterial research alongside students at their respective universities. There were three components to the BBE: a collaborative fabrication workshop with students in January 2022, full-scale installations outdoors on the UVA grounds, and an accompanying indoor gallery exhibition at the UVA School of Architecture. An opening symposium fostered discussion between the organizers and exhibitors on how renewable, carbon-sequestering biomaterials could be utilized in contemporary construction, while establishing a multi-institutional scholarly discourse that raised public awareness of novel biomaterial construction.

**Figure 5:**
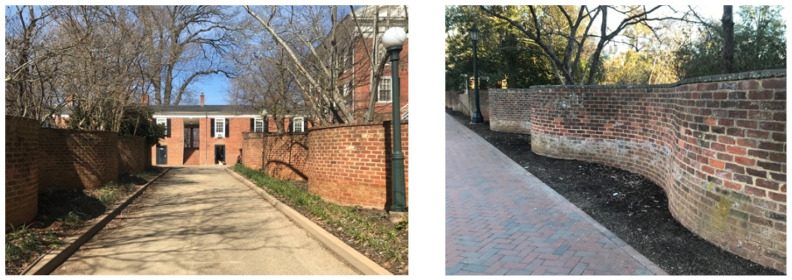
Serpentine walls designed by Thomas Jefferson and built by slave labor that enclose the gardens at the rear of the residences of the historical academical village at the University of Virginia located in Charlottesville, VA, USA. Photos by the author.

The UVA’s academical village (now a UNESCO World Heritage Site) provided potent inspiration behind the geometry of the two prototype structures presented in this paper. Established and designed by Thomas Jefferson, a prominent feature of the grounds are the brick “serpentine” walls (Figure 5) enclosing the gardens behind each residence pavilion. The structures served as barriers to between the enslaved people and the white university community and to mask the use of slave labor visually and acoustically [55]. The history of serpentine walls at the UVA and their connection to slavery is inescapable. However, serpentine walls are not Jefferson’s invention. Straight masonry walls, unless very thick or reinforced, cannot resist lateral loads [56]. Undulating walls can have a much wider footprint, which helps resist lateral loads and can be much thinner than straight walls. Therefore, the motivation to deploy serpentine wall technology for the BBE was to recontextualize the serpentine geometry from its connections to slavery. The prototypes intended to foreground that the inherent properties of myco-materials can through their flexibility, stability, and material efficiency, also act as a means of promoting environmental and human justice.

### 2.1. Parametric Design for Serpentine Walls

**Figure 6:**
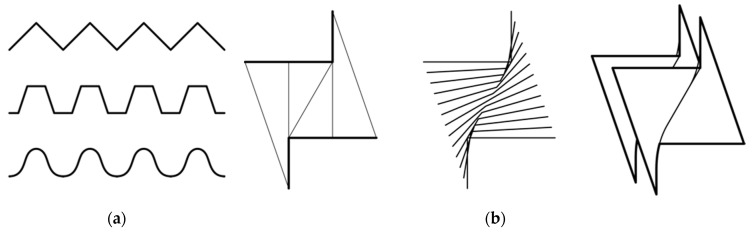
(**a**) Examples of poly-line- and poly-curve-based periodic curves generated with the parametric design script for designing serpentine walls; (**b**) three-dimensional extractions and transformations afforded by the script. Vertical slicing and thickening were both used to design formwork schemes and to estimate material volume requirements.

To facilitate the generation of an expansive and diverse family of undulating wall geometries, a custom computational design script was developed in Rhino/Grasshopper [57]. The script generated a range of three-dimensional forms for serpentine walls. The geometries were generated from a periodic base curve that informed later design decisions, including the generation of digital fabrication protocols for making the formworks. First, a curve was generated using design variables that included the number of control points that composed a V or U-shaped “unit”, the length and width of the unit, whether the curve was generated with poly-lines or poly-curves, and how many units composed the length of the curve. Figure 6a shows three examples of such basic walls in top view. Next, the underlying three-dimensional geometries of the wall units were represented as a planer ruled surface. The geometries used for the two prototypes were generated from the base curve and its mirror, according to a specified height. The final design stages consisted of a set of thickening and geometrical extraction protocols (Figure 6b) that helped generate the formwork schema specific to the myco-fabrication technique being tested.

### 2.2. Summary of Myco-Fabrication Methods

The experimental structures grown for the biomaterial building exposition tested myco-welding and fabric-forming techniques for growing large monolithic blocks and assembling them into efficiently formed wall structures. Due to their inherent lightness, assemblies of large elements were not only possible, but also offered potential advantages over other previously demonstrated methods of building with myco-materials. Taking inspiration from pre-cast concrete traditions, the goal for both prototypes was to demonstrate re-usable formwork systems that produced large myco-material building components offsite in semi-controlled working conditions. The strategies intended to reduce the demand of long labor hours, reduce the risk of contaminating large colonies of myco-materials, and reduce uncertainty during on-site assembly. Numerous practical and contextual considerations had to be determined, which ultimately influenced the specific designs and techniques used to complete them. These considerations included if the structure was going to be exhibited indoors or outdoors, the location the structure’s parts were going to be grown, the materials and fabrication resources on-hand for fabricating the formwork, how many students were available to contribute to the project, and if the author would be present for the various stages of growing and assembly. While the two structures shared a common underlying formal logic for undulating “serpentine” walls, the formal character and complexity of each serpentine wall prototype was intimately related to its respective method of myco-fabrication. The two structures were grown in two different geographic locations in the United States. At their core, these were academic projects, whereby the complexities in the form and technique had to remain accessible to UVA and K-State students both at undergraduate and graduate levels.

#### 2.2.1. Myco-Welding Slabs into Monolithic Building Units

The structure exhibited outdoors on the grounds was intended to be cultivated and assembled locally by UVA students. As such, the scale of the structure, the complexity of its form, and the accessibility of the fabrication and growing techniques were precisely selected. As a base technique, myco-welding offered numerous advantages that better aligned with the number of students involved and how much time they could contribute. In devising the proposed methodology, a driving consideration was that most of the physical effort was during a week-long workshop with participating UVA students with design and engineering backgrounds. Myco-welding was advantageous in this context because the two phases of growth de-concentrated continuous labor hours needed for large in situ monolithic mycelium casting techniques.

**Figure 7:**
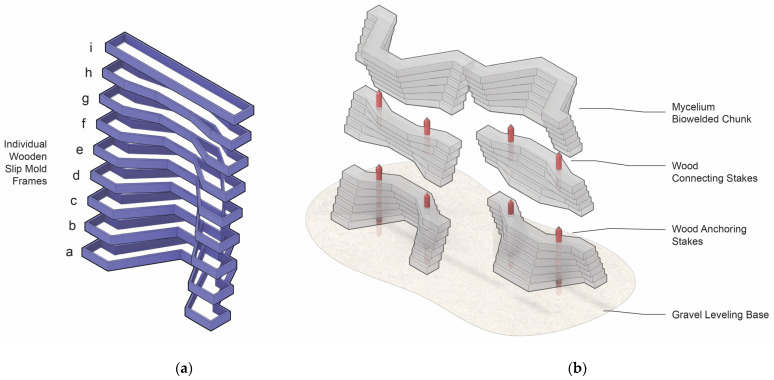
Design diagrams for La Parete Fungina. (**a**) Diagram of the nine different wooden slip-form frames (a–i) designed for growing slabs; (**b**) axonometric assembly diagram of the unit chunks highlighting the friction-based connection system of the wooden stakes. Drawings by Emmett Lockridge.

The prototype wall structure, later named La Parete Fungina, was created from two wall units, each built from seventeen V-shaped slabs myco-welded into three “chunks”. Nine different V-shaped formworks were needed (Figure 7a). Noting the labeling scheme in the figure, the palindromic sequence <a, b, c, d, e, f, g, h, i, h, g, f, e, d, c, b, a> described a complete wall unit, with eight of the forms being repeated in each unit. The formworks were intended to function as re-usable slip-molds to address the ethical dilemma of plastic or other non-cellulosic material being ubiquitously used in the production of myco-material objects. These formworks were intended to be simple to make, and because they had very limited contact with growing materials, they could be created from wood and fabricated with basic tools. For assembly on-site, a friction-based connection system (Figure 7b) was developed, so the structure could easily be disassembled when the Exposition was taken down. A unique byproduct of prolonged growth inherent to myco-welding was that it produced overgrowth: a thick layer of pure mycelium grew on the surfaces of the units. The overgrowth was like a layer of hydrophobic defense for the fungal colony, both while it was alive and after being dried and immobilized. Having such a performative benefit meant that myco-welding was the logical choice for a structure being exhibited outdoors.

#### 2.2.2. Fabric-Forming Monolithic Units

**Figure 8:**
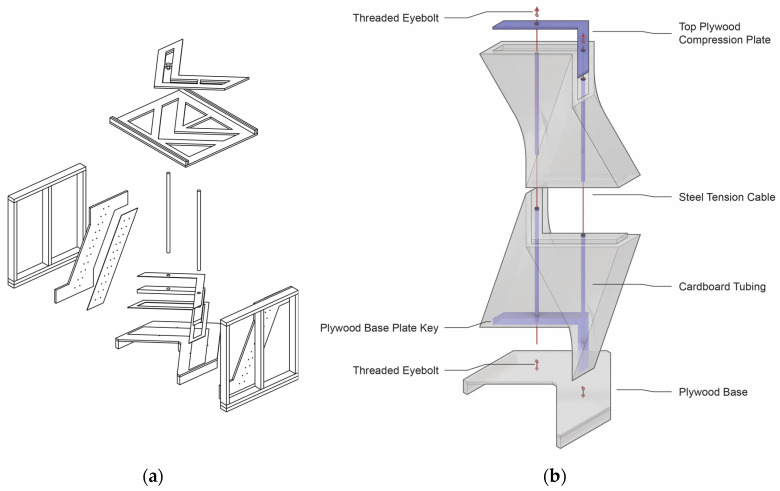
Design diagrams for L’Orso Funigno. (**a**) Exploded axonometric diagram of the formwork apparatus created to grow the monolithic wall units; (**b**) axonometric diagram of the monolithic wall units highlighting the post-tension system that applied a compressive force on the units with a mechanically tightened cable between plywood base and top plate. Drawings by Emmett Lockridge.

The structure exhibited indoors in the UVA’s School of Architecture gallery was grown in spring 2022 at K-State in the context of a research seminar instructed by the author on myco-materials and myco-fabrication. In the introduction of the course, the graduate architecture students were immersed into the question of myco-fabrication techniques for large monolithic blocks assembled into efficiently formed wall structures. They were challenged to work collaboratively and contribute efforts toward an alternative expression of myco-fabrication. In contrast to the structure being grown at the UVA, the prototype later named L’Orso Fungino leveraged the lightweight properties of myco-materials using large monolithic elements that were cast in re-usable wood and fabric formwork. The complexity and fabrication methods chosen were tuned to the available resources, the skill levels of the students involved, and the short 6-week timeline for all the design and production. In situ monolithic mycelium casting techniques were deemed advantageous, because most of the physical effort available from participating students and research assistants in the lab was during a weekly four-hour session. The custom formwork apparatus for growing the wall units (Figure 8a) was designed to be quickly assembled, collapsible, and re-usable. Vertical perforated cardboard tubes were grown into the matrix of the units to provide air into the thickest parts of the colony during growth and to later serve as a conduit for a post-tension connection system (Figure 8b). The top and bottom surfaces of each unit were detailed such that there were interlocking adjacencies between the two units, the top compression plates, and the base. Due to the anticipated lightness of each unit, a post-tensioning system was a key feature of this prototype. It was hypothesized that loading the units in compression with cables running through the cardboard tubes would bring additional strength and stability to the assembly.

### 2.3. Materials

The myco-materials used for both prototypes presented below were procured from Ecovative [13] and paid for with funds provided by the exposition. Within the budget, each prototype structure could be grown from at most one pallet of myco-materials, weighing roughly 325 kg. For these prototypes, one pallet held sixty-five 5 kg bags or 0.6 cubic meters in total volume of wet living material. Ecovative’s patented material was a hemp substrate inoculated with a fungus from the phylum Basidiomycota, whose fruiting bodies resembled the brackets produced by reishi.

The storage of these materials could have been a major challenge, because they had to be kept at approximately 4 °C to prevent the fungi from growing too quickly and fully consuming the substrate. Ideally, they should have been freight-shipped in refrigerated containers and if proper refrigerated storage was not available, immediately processed and packed into formworks. If kept unrefrigerated, the material would grow into a hardened mass in the bags within 3 to 4 days, making it labor- and time-intensive to break the hemp fibers apart. The structures grown at the UVA and K-State were grown in schools of architecture, which did not have access to large-scale refrigeration.

## 3. Results and Discussion: Two Serpentine Wall Prototypes

Each structure tested assembly strategies for growing large mycelium building units and assembling them into prototypes of efficiently formed serpentine wall prototypes. As a pair, they demonstrated the flexibility and facility of myco-materials to adapt to different approaches of fabrication based on the available tools, materials, and knowledge. La Parete Fungina demonstrated two undulating wall units standing side-by-side, each created from seventeen myco-welded slabs. L’Orso Fungino revisited the in-situ monolithic fabric forming of units that were repeated, stacked, and post-tensioned. While developing the two techniques, a major concern was to design the formwork systems to be re-usable. Consequently, the formal character and complexity of each structure were intimately related to their respective method of myco-fabrication. Both were assumed to be compression-bearing structures, even if they were not exhibited resisting external loads.

### 3.1. La Parete Fungina

**Figure 9:**
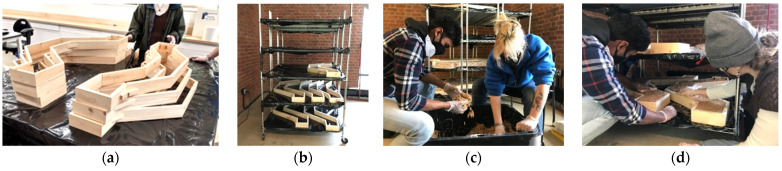

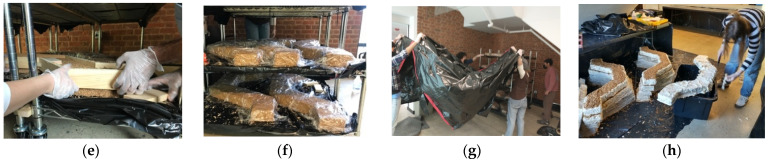
Preparation and process for forming and growing myco-material slabs: (**a**) re-usable wood formworks created in the first days of the workshop; (**b**) growing cart in its space under a staircase in the school of architecture; (**c**) fiberizing the living hemp substrate and mixing in additives prior to packing the formwork; (**d**) hand packing the wood formwork directly on the grow cart; (**e**) removing the mold for reuse; (**f**) slabs individually wrapped in food-safe plastic to keep the fibers humid and warm; (**g**) plastic “cloak” which covered the entire grow cart to keep growing specimens dark warm and humid; (**h**) myco-welding slabs with loose inoculated substrate as mortar. Photos by the author and Leila Ehtesham.

For the structure grown by UVA students, pre-emptive planning took place two weeks before the workshop in January 2022. This included the development of the script described above in Section 2.1, hiring and coordinating with a research assistant at the UVA, and purchasing materials for the formwork. The five-day workshop was attended by undergraduate students from both engineering and design backgrounds during daily four-hour sessions. For the first three days of the workshop, the primary goals were fabricating the wooden formwork and creating a growing cart. Nine wooden formworks (Figure 9a) were hand-built from 17 mm unfinished whitewood boards, cut into 75 mm strips. Each slab was 1200 mm long end-to-end and had a common rectangular cross-section 150 mm wide and 70 mm thick. The underlying poly-line V-shape of this structure produced nine formworks whose forms lay between a V and a rectangle, required measuring several non-orthogonal cuts with varying angles. This was a minor technical challenge, but was time consuming and would have benefited from digital fabrication resources. Grow-space was limited too; no more than 3 m × 3 m under a staircase. A moveable growing cart (Figure 9b) was improvised using three heavy-duty wire shelves, plastic zip ties, and black plastic sheeting. The cart had five 1220 mm × 1370 mm shelves, resulting in approximately 8.3 square meters of growing surface.

The final two workdays during the workshop were used to form and start growing the slabs. First, the inoculated hemp substrate had to be broken up until the fibers were completely loose (Figure 9c). As it was being fiberized, 250 g of kitchen flour was mixed for each 5 kg bag of living substrate. The flour was recommended by the manufacturer as a nitrogen-rich nutrient to promote the rapid growth of fungal hyphae, but for this project, the recommended quantity was doubled. The intention was to have the flour act as a temporary binder while the mycelia formed their bonds between fibers. Water was added to the extent that when a handful of fibers were squeezed, only one drop of water was released. Once fully prepared, the loose fibers were compacted by hand into the formworks on the plastic lined shelves of the cart (Figure 9d), and the wood formwork could be carefully slipped off, and reused to form multiples of the same shape (Figure 9e). As a means of providing a clean and humid environment to each slab, they were covered in food-safe plastic film (Figure 9f). The cart was covered with a black plastic covering (Figure 9g) that kept the slabs in the dark while they grew, and, more importantly, provided a second means of keeping a clean and humid growing environment. Approximately one-third of the bags were used with two days of delivery to grow a first round of slabs. During the first week-long grow period, the students stored the remaining bags in a covered outdoor space, stacked on shelves and wrapped in black plastic sheeting. This was the best option due to the lack of access to large-scale cold storage. The bags encountered temperature swings between roughly −2 and 18 °C between day and night.

In the weeks that followed the workshop, the UVA research assistant and one of the participants continued to form and grow the remaining slabs while also myco-welding the slabs that were sufficiently cultivated. The living slabs were stacked into “chunks” between five and six layers thick, with loose substrate in between to level the assembly (Figure 9h). The assembly of living parts had to be completed in rigorously clean conditions, while keeping the assembly in the correct and intended configuration. While the slabs were being stacked, they were gelatinous and fragile and had to be handled with care by at least two people at a time. Furthermore, while slabs bonded and grew together, they needed to be kept in an appropriately clean, dark, warm, and humid environment. The wall chunks were grown into monolithic-like masses for roughly two weeks, after which they were passively dried until installation. 

**Figure 10:**
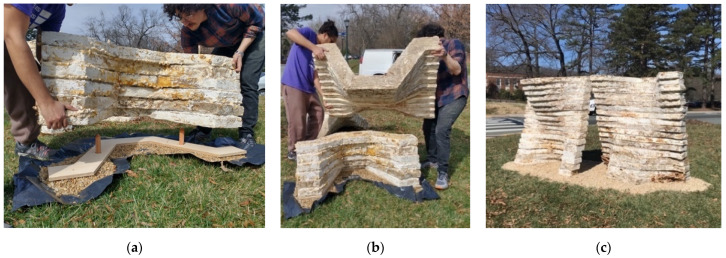
Installation of the prototype onsite: (**a**) friction fitting base chunk to the anchoring stakes in the ground; (**b**) friction fitting upper chunk of the myco-welded wall; (**c**) complete structure as it was exhibited at the biomaterials building exposition. Photos by the author and Leila Ehtesham.

At the time of assembly, all the myco-welded chucks had at minimum one week of drying time. The on-site installation of La Parete Fungina was completed in approximately one hour. The six wall chunks were driven to the site in a small passenger van. Wooden anchoring stakes (40 mm diameter) were driven into the ground and the base chunk was friction-fitted in place (Figure 10a). The remaining chunks were dry stacked (Figure 10b) each with similar wooden stakes in between. The simple friction-fitting system was ideally suited for this application, because the exhibition was temporary and needed to be taken down after a period of three months with minimal impact or damage to the UVA grounds. The two undulating units (Figure 10c) were approximately 1200 mm tall, configured in a manner such that the serpentine wall geometry produced a gap in the wall. The myco-welded objects were highly didactic due to their long growing time shown through artifacts such as changes in color to the formation of fruiting bodies. In the days following the installation, the structure was subjected to wind and snow, which did not cause a collapse, nor was the material compromised. The thick layer of overgrowth demonstrated its inherent resilience that would be well suited to its exhibition outdoors for roughly two months, after which it was taken down.

### 3.2. L’Orso Fungino

**Figure 11:**
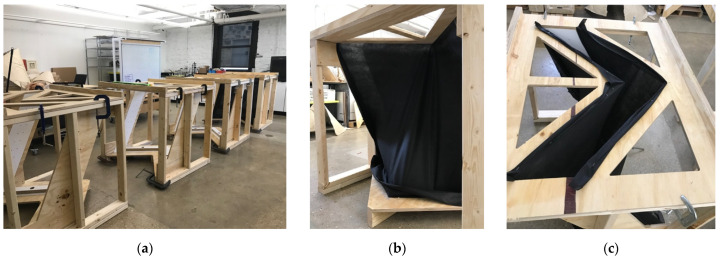
Fabric formwork apparatuses: (**a**) rigid elements of the apparatus composed of wood and plastic laminate sheet where there would be contact with living materials; (**b**) upholstered formwork with black synthetic geotextile fabric; (**c**) top of the formwork apparatus showing the geotextile upholstered into the rigid frame. Photos by the author.

For the structure grown at K-State, pre-emptive planning took place for two weeks following the week-long workshop at the UVA. With the change in strategy for growing the wall chunks, notable adjustments to the scale and geometry were determined. An important driver was that the structure needed to be shipped 1800 km from Manhattan, Kansas, to Charlottesville, Virginia. Within the budget, two pallets could be sent (1220 mm × 1016 mm in area for each). Sized according to the freight limitations, wall units were designed, each approximately 750 mm long and 750 mm tall. The prototypes intended to demonstrate the wall units was vertically stacked in twos. The formwork was designed to be quickly assembled, collapsible, and re-usable. The rigid portion of the apparatus (Figure 11a) was created with a combination of hand-cut nominal timber frames, CNC-cut plywood panels, and 3 mm plastic laminations for surfaces in direct contact with living materials. The hand-stretched fabric portions (Figure 11b,c) were composed of breathable synthetic geotextile. Several formworks were fabricated so that multiple units could be grown simultaneously.

The first fabric formwork apparatuses were packed roughly one month before the opening of the exposition. Within the time constraints, two units could be packed by six people. As a safeguard from potential failures, additional units were accounted for in the materials budget. Due to a limited supply of materials, it was decided that non-sterilized and non-inoculated hemp fibers would be mixed in with the inoculated substrate to increase the yield volume. Several previous experiments in the MycoMatters Laboratory successfully propagated Ecovative materials into ratios of up to one part inoculated to four parts non-inoculated and non-sterilized hemp fibers (1:4) by volume. The fabric formworks were packed with a 1:2 ratio to increase the volume with less risk. In addition to the hemp, 250 g of kitchen flour per 5 kg bag of living material and water was added such as above.

**Figure 12:**
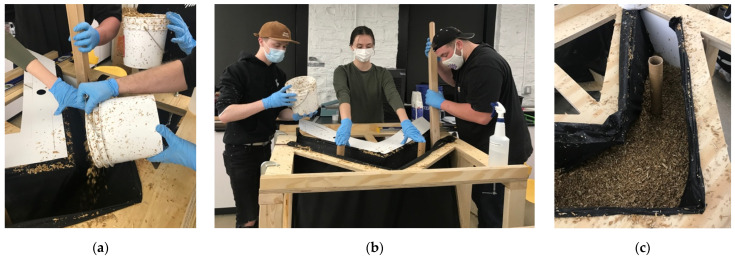
Packing the fabric formwork apparatus with living myco-materials: (**a**) loose inoculated substrate was poured into the apparatus; (**b**) material was compressed into the form while minding the cardboard conduits; (**c**) apparatus almost full of inoculated substrate. Photos by the author.

**Figure 13:**
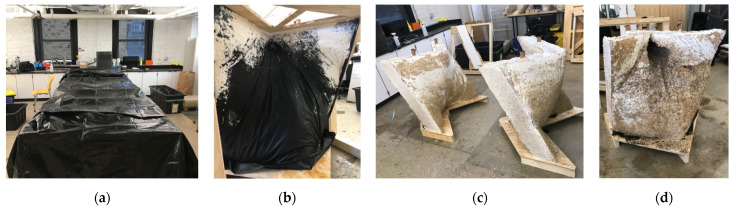
Growing fabric-formed monolithic wall units: (**a**) black plastic covered the formworks as the material grew in the MycoMatters Laboratory; (**b**) after four days of growth, healthy mycelium was found growing through the fabric; (**c**) first two fabric-formed monolithic units with all formworks removed after growing four days; (**d**) third unit found with a contamination compromising mycelial growth deep into the unit. Photos by the author.

**Figure 14:**
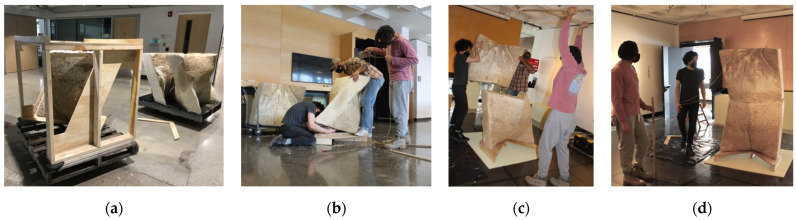
Assembling the prototype: (**a**) palletized units and formwork apparatus before shipping to the UVA; (**b**) stringing cables from base plate through lower wall unit; (**c**) stacking two wall units while pulling tension cables through the assembly; (**d**) stacked undulating wall units before post-tensioning. Photos by the author and Leila Ehtesham.

Within a four-hour work period, two formworks were filled with inoculated substrate (Figure 12a). The formworks were packed monolithically by hand and with the help of tools to compress the material around the perforated cardboard tubes (Figure 12b). Each unit used between nine and ten bags of pre-inoculated material due to overpacking, which caused the fabric to stretch, ultimately requiring more material to fill the formwork (Figure 12c). The formwork was then covered with a black plastic covering that kept the material humid and dark while the mycelium grew (Figure 13a). After the first two units were packed, a third was packed a day later. During the growth period of the first three units, approximately fifteen bags could be stored in the lab refrigerator. The remaining twenty bags (approximately) had to be kept on a pallet in the lab. After only four days, mycelium from the first two units had already grown through the stretched fabric (Figure 13b). The formwork of the first two units was removed (Figure 13c), which meant they would have two full weeks to passively dry in the lab before being shipped. The third unit became contaminated (Figure 13d) deep in the monolithic colony, despite that surface mycelium managing to grow in many areas. This suggested that a contaminant from the non-sterilized hemp and a lack of air were probably contributing causes. The first two formwork apparatuses were re-assembled and re-used to grow two more units. Of those two, one more unit became contaminated. The remaining three fabric-formed monolithic units and a wooden formwork apparatus were palletized (Figure 14a) and shipped to the UVA.

**Figure 15:**
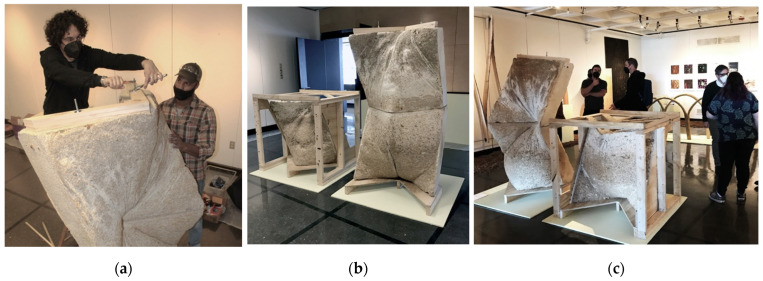
Post-tensioning and exhibiting the prototype: (**a**) tightening the internal cables to add compressive force to the assembly; (**b**) gallery installation with stacked units and wood formwork; (**c**) wall prototype during exhibition opening at the UVA. Photos by the author and Leila Ehtesham.

The on-site installation of L’Orso Fungino was completed in approximately two hours. The assembly of the prototype began by stringing cables through cardboard conduits of the lower wall unit (Figure 14b). The steel cables were mechanically fastened to the plywood base compression plate. Next, the cables were strung through the top wall unit (Figure 14c), this time with more difficultly because one of the cardboard tubes bent during the packing process. None of the units was fully dry which was an advantage because the cable could be forcefully pushed through the spongy mycelial matrix. Cables were mechanically fastened to threaded eyebolts. The stack of two units (Figure 14d) had a top plate that was put in compression by the tightening of each eyebolt against a washer (Figure 15a). The assembly was allowed to compress by one centimeter from post-tensioning. L’Orso Fungino was exhibited in the gallery at the School of Architecture at the UVA, alongside the third wall unit and the formwork apparatus (Figure 15b,c). The post-tension system was successful as a technique for stabilizing myco-structures. The connection details are in need of future iterations. A larger open question was how such post-tensioned wall structures would support the load of a vault, a truss, or a beam. In its current state, the post-tensioning system was simple to disassemble at the end of the exposition.

### 3.3. Discussion and Future Work

La Parete Fungina and L’Orso Fungino both served as demonstrations of wall assembly systems that challenged the status quo of myco-fabrication. Myco-welding and fabric-forming techniques were tested for their capacity to create complex yet efficiently formed wall structures from large building units grown in re-usable formwork systems. The structures initiated a new dialog of architecture-scale myco-fabrication techniques that were positioned between those which used building units the size of a brick and those which used units the size of a room. They demonstrated the flexibility and ease with which myco-materials could adapt based on available tools, materials, and knowledge.

Working in educational contexts, the strategies generated material knowledge directly through the production of physical artifacts. For novice student collaborators, this approach was productive toward fostering their appreciation and mastery of building material assemblies through the technical lens of myco-materials. Furthermore, students had the freedom to exercise their creativity and experience working at full-scale through experiments that left room for improvised adjustments. Insights on the craft of growing myco-structures were never assumed, were developed directly in collaboration with the students, and generated through creating. The students learned first-hand that building (with any material) is challenging, but also joyful and rewarding. Thus, the impacts of the methods presented below were both technological and educational. Myco-materials were challenging for students because they required greater care and attention compared to common, inert materials. For example, compared to handling live materials, personal protection practices, including wearing masks and gloves and thoroughly cleaning all working surfaces and formwork, are not an inherent protocol for a typical design or engineering student. Lessons learned through minor frictions, failures, and contaminations were vital to provoke important questions about the material ethics and the appropriateness of the methods.

Rather than demonstrate methods ready to grow buildings entirely from myco-materials the prototypes suggested new hybrid techniques that could contribute to the broader cannon of myco-fabrication; adding to well-established discrete element, 3D printing, and monolithic techniques. The prototypes detailed above operated between the scales of a brick and a monolithic pavilion. At the scale of a house (for example), a building structure grown from myco-materials would likely require multiple prefabricated units or “chunks” that would be assembled on-site. Without commercial-scale growing resources, there are practical and biological limits to the scales of colonies one can grow. The Monolito Micelio [47] was 2.5 m × 2.5 m × 2.5 m, and its scale presented several notable disadvantages, including the significant demand for time and labor, the risk of handling such significant quantities of living material in uncontrolled environments, the need for pliable internal reinforcing, and the challenge of drying large colonies to stop growth without large ovens. While there were few active energy inputs needed to grow the myco-materials, the near-ubiquitous plastic formworks in which they were grown presented an ethical dilemma. The plastic-lined plywood and woven nylon fabric formwork system that formed the Monolito Micelio were a waste byproduct that resulted in landfill disposal. However, if a sufficient volume of living myco-materials was available and there was enough funding to hire labor for such a project, most of the formwork could have been reused to create several pavilion-scale units that could be aggregated to form larger spaces. This suggested that the techniques demonstrated by the Monolito Micelio could be scaled to grow parts two to four times larger. The goal for myco-fabrication in architecture should be to develop the capacity to grow parts at the same scale as current glue-laminated and cross-laminated timber manufacturing.

Experimentation with myco-fabrication at architectural scale is still nascent as a field and faces massive challenges that need to be overcome to achieve results other than prototype structures and fanciful pavilions. Limitations for building-scale deployments of myco-materials are foremost caused by the challenge of supply and access to commercial quantities of material. Currently, the lack of myco-material production infrastructure makes it both energetically and financially costly to transport wet, living mycelium composites over long distances. Ecovative, which is based in Troy, NY, has pioneered the scaling-up of myco-material production by closely studying and collaborating with commercial mushroom farming industries, especially in the neighboring state of Pennsylvania, where over 60% of all mushrooms in the United States are grown [58]. As a result, they offer a biotechnology that grows quickly and reliably under favorable environmental conditions and with some resilience to contamination. The ability to grow mycelium entirely through the lignocellulosic substrate within just a few days is a major advantage that makes growing structures a relatively fast process. However, such speed and reliability come with costs that can be a barrier to using myco-materials at a large scale. Beyond the cost of the material itself, the need to hire expedited refrigerated transport and have access to large-scale refrigerated storage are other potential barriers.

Radical approaches to sustainable construction raise questions of material ethics when evaluating what and how much was wasted in different approaches to myco-fabrication. Although in many regional contexts myco-material technologies have the capacity to require less energy than petrochemical foams and plastics they seek to replace, there are critical ethical questions that must be considered. In addition to the near-ubiquitous use of plastic and issues of waste-producing formworks, transporting myco-materials long distances with petroleum-consuming vehicles should be scrutinized. For the prototypes presented here, live myco-materials were procured from Ecovative’s spawn supplier in Pennsylvania and transported either to the UVA or K-State. For both, it was cost-prohibitive to hire a refrigerated truck to deliver from the spawn supplier directly to the university. Thus, more improvisatory and self-motivated methods were needed. For the UVA-grown structure, shipping delays were going to have the material arrive after the workshop was over. To keep the project on schedule, the author drove a 750 km round-trip to the spawn supplier. For the structure grown at K-State, creative logistical planning was required to organize a two-step relay delivery. For a pro-rated cost, the pallet was first “hitch-hiked” 1700 km with a scheduled refrigerated shipment of button mushrooms spawned on a farm in the neighboring state of Oklahoma. Research assistants in the MycoMatters Laboratory then drove an 800 km roundtrip to bring the pallet back from the farm. It would be ideal for the distribution of myco-materials to exist in a model where they are regionally produced as a way of reducing transportation distances. More immediately, the opportunity to overlay the myco-material demand with existing food supply chain maps from existing spawn suppliers presents an opportunity to cultivate deep bonds between agriculture and biomaterial industries.

For myco-materials to succeed in the future, they must remain in the current dialog, both in academic and professional contexts. Collaborations across the fields of design, engineering, biological sciences, agriculture, and economics (for example) are essential to developing new knowledge essential for the scaling of new material technologies in ways relevant to building construction. Importantly, knowledge about growing structures must be as commonly accessible such as knowledge needed for building with wood, steel, or concrete. The craft of growing myco-structures is clearly in need of time to mature, but will only do so through continued study and experimentation. Future work should seek cooperative logics between fungal growth, computational design, and digital fabrication to further discover constructive possibilities with myco-materials. For example, digital reference technologies, such as 3D-scanning and augmented reality, may be important components of future work on myco-welding. The ability to grow twine and other natural-fiber textiles into the material matrix suggests that through computational design and analysis, the strategic placement of such reinforcements could be deployed to selectively strengthen and enhance myco-materials. Such advancements could help fully integrate forming materials into the building components being grown. Pre-stressing and post-tensioning have probable futures in myco-fabrication for certain structural scenarios. Robotic fiber winding and CNC knitting are two technologies that have been widely demonstrated and could be immediately applied in the context of myco-fabrication. The raising popularity of “co-bots” also suggests a future in which machines could collaborate with craftsmen and carry out improvisational tactics with greater precision, reduced demand for labor, and potentially much safer and cleaner fabrication conditions.

## 4. Summary and Conclusions

There is an urgent need for low-energy and renewable building materials that divert building and demolition waste from landfills and lessen the impact of the construction industry on climate change. The ability to rapidly grow building structures from myco-materials, particularly for short-term or temporary functions, has the potential to greatly reduce building and demolition waste. This paper provided an extensive overview of the state-of-the-art in deploying myco-materials at the architectural scale, highlighted the numerous case studies of researchers in diverse global contexts growing building-scale structural parts and pavilions, and gave first-hand insight about the significant challenges and limitations associated with the application of myco-materials for architectural structures. The applied research presented here developed hybrid myco-fabrication techniques that overcame the challenge of cultivating large colonies of living myco-materials into precise forms, and demonstrated intuitive and re-usable formwork systems that reduced waste byproducts from growing and fabrication processes. The techniques were developed in academic environments that gave young designers and engineers the access, space, and resources for working with myco-materials. The two prototype wall structures demonstrated the ability to grow large and complex shapes outside of rigorously controlled biolab environments, and with fewer risks than monolithic structures grown in situ. The lightweight properties of mycelium composites were an advantage in this context, where large, complex building components could be pre-grown and pre-dried off-site in semi-controlled environments and assembled with less continuous on-site labor compared to the production of brick/block or monolithic mycelium structures.

Realistically, the greatest potential for these techniques is in applications that replace EPS and other varieties of foam and insulation materials for insulated concrete formworks, large-scale acoustical arrays, temporary self-supporting structures, interior furnishings, scenography or theatre stage projects, and others, leveraging the inherent absorptive, insulative, and fire-resistant properties of myco-materials. Whether these techniques are applicable to load-bearing building structures is still an open question that demands further research. Nonetheless, large-scale building and long-span myco-material structures continue to gain interest in trends of research and commercialization that seek to vastly offset the impact of the short lifespans of buildings in the modern era.
